# NLRP3 inflammasome activation in platelets in response to sepsis

**DOI:** 10.14814/phy2.14073

**Published:** 2019-05-03

**Authors:** Denise C. Cornelius, Cedar H. Baik, Olivia K. Travis, Dakota L. White, Cassandra M. Young, W. Austin Pierce, Corbin A. Shields, Bibek Poudel, Jan M. Williams

**Affiliations:** ^1^ Department of Emergency Medicine University of Mississippi Medical Center Jackson Mississippi; ^2^ Department of Pharmacology University of Mississippi Medical Center Jackson Mississippi; ^3^ Cardiovascular Renal‐Research Center University of Mississippi Medical Center Jackson Mississippi

**Keywords:** Endothelial permeability, inflammation, multiorgan injury, NLRP3, platelets, sepsis

## Abstract

Sepsis is a complex syndrome characterized by organ dysfunction and a dysregulated immune host response to infection. There is currently no effective treatment for sepsis, but platelets have been proposed as a potential therapeutic target for the treatment of sepsis. We hypothesized that the NLRP3 inflammasome is activated in platelets during sepsis and may be associated with multiorgan injury in response to polymicrobial sepsis. Polymicrobial sepsis was induced by cecal ligation and puncture (CLP) in 12‐ to 13‐week‐old male Sprague–Dawley rats. The necrotic cecum was removed at 24 h post‐CLP. At 72 h post‐CLP, activated platelets were significantly increased in CLP versus Sham rats. Colocalization of NLRP3 inflammasome components was observed in platelets from CLP rats at 72 h post‐CLP. Plasma, pulmonary, and renal levels of IL‐1*β* and IL‐18 were significantly higher in CLP rats compared to Sham controls. Soluble markers of endothelial permeability were increased in CLP versus Sham. Renal and pulmonary histopathology were markedly elevated in CLP rats compared to Sham controls. NLRP3 is activated in platelets in response to CLP and is associated with inflammation, endothelial permeability and multiorgan injury. Our results indicate that activated platelets may play a role to cause multiorgan injury in sepsis and may have therapeutic potential for the treatment of sepsis multiorgan injury.

## Introduction

Sepsis is a complex syndrome characterized by organ dysfunction and a dysregulated immune host response to infection (Greco et al. 2017). Septic patients are particularly at risk for the development of acute lung injury (ALI) and acute kidney injury (AKI). Impaired organ function is the primary common clinical manifestation of sepsis, with the lungs often being the first organ system to undergo a dysfunctional response, resulting in the development of ALI. Consistent with its role in promoting multisystem organ dysfunction, sepsis is the most common contributing factor to AKI, accounting for up to 50% of AKI cases in developed countries (Alobaidi et al. 2015; Umbro et al. 2016). Forty‐three percent of sepsis‐associated death is caused by multiple organ failure (Vincent et al. 2011). Unfortunately, therapies targeting a single component in the inflammatory cascade have failed to reduce ALI, AKI, and the high mortality rate associated with sepsis. Thus, alternative and more effective approaches to treat complications of sepsis are of great clinical importance.

The contribution of platelets to sepsis progression goes beyond thrombosis and coagulation. Platelets have emerged as major drivers of the innate and adaptive immune response. Septic patients with increased platelet activation and low platelet count are prone to develop multiple organ dysfunction and have increased 90‐day mortality (Gawaz et al. 1997; Russwurm et al. 2002; de Stoppelaar et al. 2014; Greco et al. 2017; Layios et al. 2017). While we know that platelet activation is increased in sepsis patients and is positively associated with multiorgan injury and mortality, its role in the disease process remain unclear.

Our overall goal was to determine the role of activated platelets in the development of multiorgan injury in sepsis. One possible mechanism by which platelets may contribute to multiorgan injury is through activation of the NOD‐like receptor protein 3 inflammasome (NLRP3). Inflammasomes are cytoplasmic complexes responsible for activation of the proinflammatory cytokines, IL‐1*β* and IL‐18 (Abderrazak et al. 2015). NLRP3 activation is linked to a number of inflammatory conditions, including sepsis (Long et al. 2016). Inhibition of NLRP3 is protective against sepsis‐induced organ injury (Luo et al. 2014; Gong et al. 2015; Moon et al. 2015; Wang et al. 2015; Kalbitz et al. 2016) and shock (Mao et al. 2013; Gong et al. 2015). However, there have been only a few studies that have examined NLRP3 activation in platelets (Hottz et al. 2013; Murthy et al. 2017); and none have examined the role of platelet NLRP3 activation in sepsis. In this study, we hypothesized that NLRP3 is activated in platelets in response to cecal ligation‐puncture (CLP) and is associated with multiorgan injury in response to polymicrobial sepsis. We used the CLP rat model to investigate NLRP3 assembly and activation in platelets in response to in vivo sepsis.

## Materials and Methods

### Animals

Male Sprague–Dawley rats purchased from Envigo (Indiandapolis, IN) were used in this study. All experimental procedures executed in this study were in accordance with the National Institutes of Health guidelines for use and care of animals. All protocols were approved by the Institutional Animal Care and Use Committee at the University of Mississippi Medical Center. The care and handling of the animals were in accord with the National Institutes of Health guidelines for ethical animal treatment.

### Cecal ligation and puncture

All of our in vivo experiments were performed in 12‐ to 13‐week‐old male rats weighing approximately 300–325 g. The animals were randomly divided into two groups: sham operation group (Sham, *n* = 10) and cecal ligation‐puncture group (CLP, *n* = 12). Under isoflurane anesthesia the CLP surgery was performed on a subset of rats to induce abdominal polymicrobial sepsis as previously described(Hubbard et al. 2005). Preheated saline (20 mL/kg, 37°C) was subcutaneously injected immediately after the operation for resuscitation. Sham‐operated animals underwent the same surgical procedure without cecum ligation or puncture. The rats were placed back into their cages after surgery, and food and water were provided ad libitum. At 24 h post‐CLP, the prior incision was reopened and the necrotic cecum was carefully excised. The abdominal cavity was washed twice with 30 mL of warm sterile saline solution. The abdominal incision was again closed in layers. Animals were administered a broad spectrum antibiotic daily (Naxcel 5 mg/kg) and buprenorphine SR (1.2 mg/kg) to control pain over the 72 h experimental period. At the end of the protocol, the animals were sacrificed under deep anesthesia. Blood and tissues were collected for further analysis.

### Platelet isolation

Arterial blood was drawn into acid‐citrate‐dextrose vacutainer tubes and centrifuged at 200*g* for 20 min to obtain platelet‐rich plasma. Platelet‐rich plasma (PRP) was mixed with an equal volume of HEP buffer (140 mmol/L NaCl, 2.7 mmol/L KCl, 3.8 mmol/L HEPES, 5 mmol/L EGTA, pH 7.4) containing 1 *μ*mol/L PGE1. This mixture was then centrifuged at 100*g* for 20 min to pellet remaining RBC and WBC in which 40 *μ*L of the supernatant were used to count platelets. The PRP was then centrifuged at 800*g* for 20 min, the supernatant was discarded and the pellet was resuspended in Tyrode's buffer (134 mmol/L NaCl, 12 mmol/L NaHCO_3_, 2.9 mmol/L KCl, 0.34 mmol/L Na_2_HPO_4_, 1 mmol/L MgCl_2_, 10 mmol/L HEPES) containing 5 mmol/L glucose and 3 mg/mL BSA. Additionally, 5 × 10^5^ platelets from CLP and SHAM rats were plated in 3 wells of a 96 well plate in a total of 200 *μ*L of Tyrode's buffer. After overnight incubation at 37°C, the media were collected and pooled for further analysis.

### In vitro LPS stimulation

1 × 10^7^ platelets from normal Sprague–Dawley rats were plated in the well of a 6‐well plate or in chamber slides and treated with 100 ng/mL *Escherichia coli* LPS (L5024, Millipore Sigma, St. Louis, MO), 100 ng/mL recombinant LPS‐binding protein (6635‐LP‐025, R&D Systems, Minneapolis, MN), and 100 ng/mL recombinant CD14 (SRP6036, Millipore Sigma) or Tyrode's buffer only for 2 h at 37°C. Platelets were collected and prepared for flow cytometry analysis and confocal microscopy.

### Flow cytometry

Freshly isolated or LPS stimulated platelets (10^6^) were resuspended in 50 *μ*L of Tyrode's buffer. Platelets were incubated (RT for 30 min) with fluorescein isothocyanate (FITC) ‐conjugated anti‐mouse CD41 (Clone MWReg30, BioLegend, San Diego, CA) and Allophycocyanin (APC) conjugated anti‐mouse/rat CD62P (Clone RMP‐1, Biolegend). A minimum of 10,000 events per gate were acquired using a MACsQuant Analyzer 10 (Miltenyi Biotec, Auburn, CA) and analyzed using FlowLogic software (Innovai, Sydney, Australia). Platelets were distinguished by specific binding of anti‐CD41 and characteristic forward and side scattering. Platelets staining positive for CD41 and CD62P were designated as activated platelets.

### Confocal and light microscopy

Platelets (10^7^) from Sham or CLP rats or stimulated with or without LPS were allowed to adhere to permanox slides (Lab‐Tek, Thermo Fisher Scientific, Waltham, MA). Adhered cells were fixed in 10% formalin for 30 min, washed 3Xs with PBS for 10 min at RT, and permeabilized with ice cold 100% methanol for 10 min. The slides were blocked in PBS with 10% goat and rabbit serum for 1 h, washed and incubated with rabbit anti‐NLRP3 antibody (NBP2‐12446, Novus Biologicals, Littleton, CO) for 2 h at RT. Slides were then washed and incubated with anti‐rabbit Alexa Fluor 488 (A11070, Thermo Fisher Scientific) for 1 h at RT. The slides were washed again and incubated with Alexa Fluor 647‐conjugated rabbit anti‐ASC antibody (NBP1‐78977AF647, Novus Biologicals) for 1 h at room temperature. Controls were processed identically, except for omission of the primary antibodies. Preparations were mounted in Cytoseal 60 (Thermo Scientific) and analyzed on a Nikon C1 (Nikon) confocal scanning microscope. Representative light microscopy images of platelets from Sham and CLP rats are presented in Figure S1. The images were captured using a Nikon Eclipse 55i microscope equipped with a Nikon DS‐Fi1 color camera (Nikon, Melville, NY).

### Caspase 1 activity assay

Caspase 1 activity was assessed using the Caspase 1 Assay Kit (Abcam, Cambridge, MA) according to the manufacturer's instructions.

### Assessment of renal injury

Kidneys were collected, weighed and fixed in a 10% buffered formalin solution. Paraffin sections (3 *μ*m) were prepared and stained with Periodic acid‐Schiff to assess the degree of glomerular injury on approximately 30 images per section per rat. Thirty glomeruli per section were scored in a blinded fashion on a 0–4 scale with 0 representing a normal glomerulus, 1 representing a 25% loss, 2 representing a 50% loss, 3 representing a 75% loss, and 4 representing >75% loss of capillaries in the tuft. Images were captured using a Nikon Eclipse 55i microscope equipped with a Nikon DS‐Fi1 color camera (Nikon Inc.; Melville, NY) at 40× using NIS‐Elements D 3.0 software. From the terminal blood sample, the plasma concentration of creatinine (Pcr) (Wako Chemicals USA, Richmond, VA) was measured to assess renal function.

### Assessment of lung injury

Histological evaluation was performed on lungs via hematoxylin and eosin (H&E) staining. A lung lobe was formalin‐fixed. The tissue was subsequently paraffin‐embedded and 5‐*μ*m sections were stained with (H&E). Sections were evaluated using light microscopy (Kaya et al. 2015). Images were captured using the same microscope, camera, and software as mentioned previously. To quantify the magnitude of pulmonary edema, a lung wet‐to‐dry (W/D) ratio was determined. The wet weight was determined by excising the left lung lobe, blotting it dry, and then immediately weighing it. The excised lung lobe was then placed in an oven at 80°C for 72 h, and then re‐weighed to obtain the dry weight. The W/D ratio was calculated by dividing the wet weight by the dry weight.

### Quantification of biomarkers of endothelial permeability and cytokines

Plasma levels of Angiopoietin‐2 (MANG20, R&D Systems) and Endocan‐1 (MBS039900, MyBioSource, San Diego, CA) were measured as markers of endothelial permeability by ELISA according to the manufacturer's protocol. Plasma, kidney, and lung IL‐1*β* (RLB00, R&D Systems) and IL‐18 (ab213909, Abcam, Boston, MA) were quantified by ELISA according to the manufacturer's protocol. Levels of IL‐1*β* in collected platelet media were also quantified by ELISA according to the manufacturer's protocol. Kidney and lung lysates were prepared with the BioPlex Cell Lysis Kit (171304011, BioRad, Hercules, CA) according to the manufacturer's protocol. Tissue cytokine levels were normalized to protein concentration using a protein assay with BSA standards (Pierce, Rockford, IL).

### Statistical analysis

All data are expressed as mean ± SEM. Statistical analyses were performed with Student's *t* test. A value of *P* < 0.05 was considered statistically significant.

## Results

### Platelet activation after in vitro LPS stimulation or CLP

**Figure 1:**
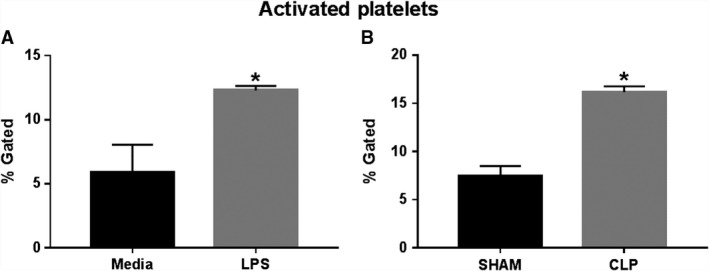
Platelet activation in response to experimental sepsis. Rat platelets were exposed to media or LPS in vitro (A; *n* = 3), or isolated from Sham and CLP rats (B; *n* = 10/group). Percentage of CD41^+^ CD62P^+^ activated platelets was quantified via flow cytometry. *P < 0.05 versus Sham.

The percentage of activated platelets after in vitro stimulation with LPS or from Sham and CLP rats was assessed using flow cytometry. LPS treatment significantly increased platelet activation versus control media (12.3 ± 0.3% vs. 5.9 ± 2.2% gated CD41^+^/CD62P^+^cells, *P* < 0.05, Fig. 1A). Similar results were observed in in vivo experiments. Activated platelets in Sham rats were 7.4 ± 1.1% gated versus 16.2 ± 0.6% gated in CLP rats (*P* < 0.05, Fig. 1B). Platelet counts decreased from 25.64 × 10^3^/*μ*L in Sham to 12.52 × 10^3^/*μ*L in CLP rats (*P* < 0.05).

### NLRP3 inflammasome assembly after in vitro LPS stimulation or CLP

**Figure 2:**
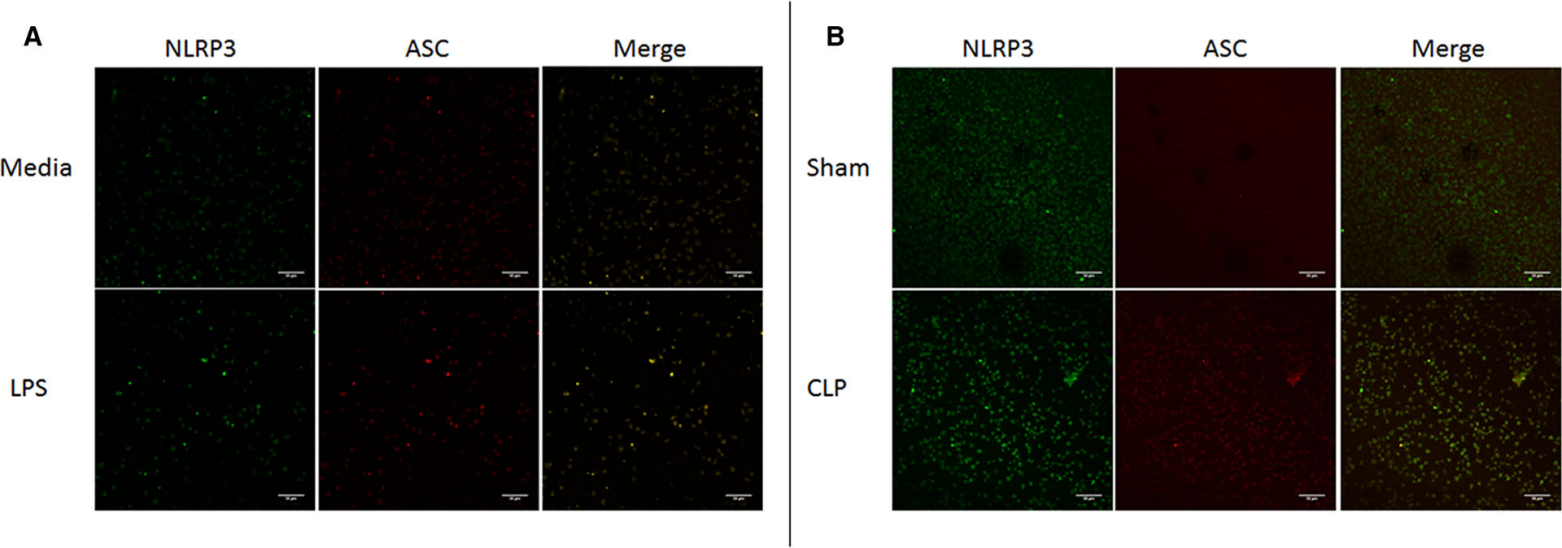
NLRP3 inflammasome assembly in response to experimental sepsis. Rat platelets were exposed to media or LPS in vitro (A), or isolated from Sham and CLP rats (B). Platelets were stained with fluorescent antibodies against rat NLRP3 or rat ASC. Colocalization of NLRP3 inflammasome components was visualized via confocal microscopy using a Nikon C1+ confocal scanning microscope. *P < 0.05 versus Sham.

Colocalization of NLRP3 and apoptosis‐associated speck‐like protein (ASC) was visualized in platelets after LPS stimulation and those isolated from Sham and CLP rats. Both in vitro LPS stimulation and CLP in rats resulted in colocalization of inflammasome components compared to nonstimulated platelets (Fig. 2A) or platelets from Sham rats (Fig. 2B).

### NLRP3 inflammasome activation in platelets in response to CLP

**Figure 3:**
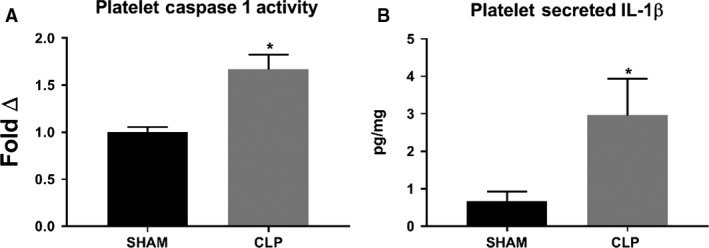
Platelet caspase 1 activity and IL‐1*β* secretion in response to CLP. Caspase 1 activity (A) was measured in platelet homogenates obtained from CLP and Sham rats. IL‐1*β* concentration (B) was measured in conditioned media from CLP and Sham rat platelets via ELISA. *P < 0.05 versus Sham.

Caspase 1 activity and secretion of IL‐1*β* by platelets were assessed to confirm activation of the NLRP3 inflammasome in platelets after induction of sepsis in CLP rats. Caspase 1 activity was significantly increased in platelets isolated from CLP rats compared to platelets isolated from Sham rats. Caspase 1 activity increased by 60% in CLP platelets compared to Sham platelets (Fig. 3A). Likewise, platelets from CLP rats secreted significantly more IL‐1*β* as compared to platelets from Sham rats. IL‐1*β* in platelet incubation media increased significantly from 0.68 ± 0.25 pg/mg in media from Sham platelets to 2.96 ± 0.98 pg/mg in media from CLP platelets (*P* < 0.05; Fig. 3B).

### Effect of CLP and NLRP3 activation on circulating, renal, and pulmonary IL‐1*β* and IL‐18

**Figure 4:**
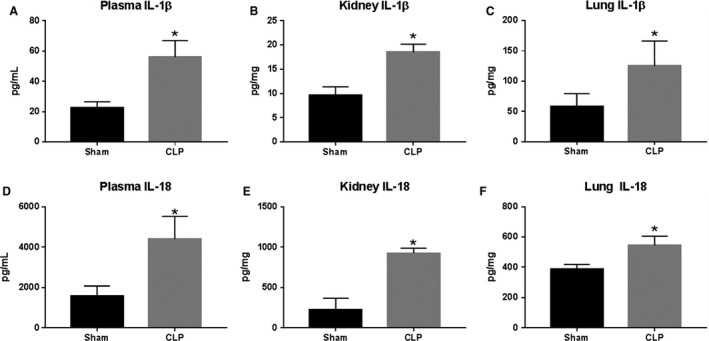
IL‐1*β* and IL‐18 expression in response to CLP. Plasma (A and D), kidney (B and E), and lung (C and F) IL‐1*β* and IL‐18 were measured in Sham and CLP rats (*n* = 8/group) via ELISA. *P < 0.05 versus Sham

Plasma, kidney, and lung homogenates were assessed for circulating, renal, and pulmonary levels of IL‐1*β* and IL‐18 using commercial ELISA kits. IL‐1*β* was significantly increased in plasma, kidneys, and lungs of CLP animals compared to Sham. Plasma IL‐1*β* was 22.7 ± 3.9 pg/mL in Sham versus 56.0 ± 10.8 pg/mL in CLP (*P* < 0.05, Fig. 4A). Renal IL‐1*β* was 9.7 ± 1.6 pg/mg in Sham and 18.5 ± 1.7 pg/mg in CLP (*P* > 0.05, Fig. 4B). Pulmonary IL‐1*β* was 58.4 ± 8.0 pg/mg in Sham and increased to 125.3±15.6 pg/mg in CLP (*P* < 0.05, Fig. 4C). IL‐18 was significantly increased in plasma, kidney, and lungs of CLP rats versus Sham. Plasma IL‐18 was 1583 ± 491.2 pg/mL in Sham and increased to 4403 ± 1114 pg/mL in CLP (*P *< 0.05, Fig. 4D). Renal IL‐18 was 220.5 ± 145.1 pg/mg in Sham and 924.2 ± 61.2 pg/mg in CLP (*P* < 0.05, Fig. 4E). Pulmonary IL‐18 increased from 387.4 ± 31.2 pg/mg in Sham to 543.7 ± 61.8 pg/mg in CLP (*P* < 0.05, Fig. 4F).

### Effect of CLP and NLRP3 activation on endothelial permeability

**Figure 5:**
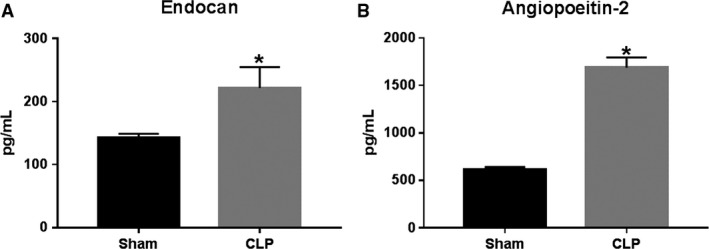
Endothelial permeability in response to CLP. Plasma levels of endocan (A) and angiopoietin‐2 (B), soluble markers of endothelial permeability were measured in Sham and CLP rats (*n* = 8/group) via ELISA. *P < 0.05 versus Sham.

Plasma levels of Endocan and Angiopoeitin‐2, markers of endothelial barrier dysfunction, were measured in animals from both groups. Endocan increased from 142.9 ± 6.0 pg/mL in Sham to 221.7 ± 33.0 pg/mL in CLP (*P* < 0.05, Fig. 5A). Similarly, Angiopoeitin‐2 increased from 617 ± 23.1 pg/mL in Sham to 1688 ± 107.1 pg/mL in CLP (*P* < 0.05, Fig. 5B).

### Effect of CLP and NLRP3 activation on multiple organ injury

**Figure 6:**
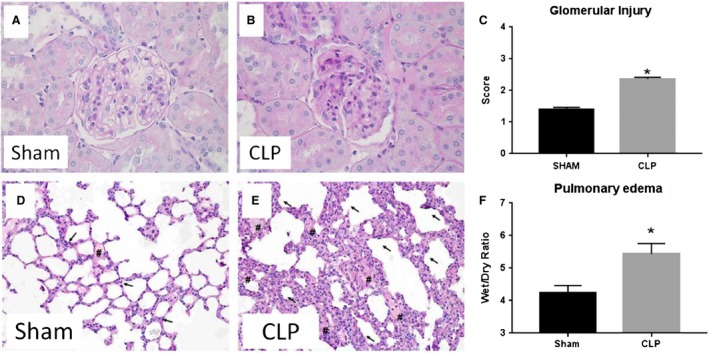
Multiple organ injury in response to CLP. Periodic Acid‐Schiff (PAS) staining was performed on paraffin‐embedded kidneys from Sham (A) and CLP (B) rats. Glomerular injury score (C; *n* = 8 rats (30 glomeruli/rat)) was scored from PAS stained samples. Hematoxylin and eosin (H&E) staining in Sham (D) and CLP (E) rats demonstrating alveolar flooding (#) and leukocyte infiltration (arrows) into pulmonary tissues. Lung wet/dry weight ratio (F) in Sham and CLP rats. *P < 0.05 versus Sham.

The effects of CLP on the degree of renal and pulmonary injury are presented in Figure 6. The kidneys from CLP rats displayed increased mesangial expansion compared to Sham rats (Fig. 6A and 6B). The glomerular injury score in CLP rats averaged 2.4 ± 0.2, indicating that greater than 50% of the glomerular capillary area available for filtration was lost (Fig. 6C). Changes in lung histology of Sham and CLP rats were also examined. As shown in Figure 6D, lungs from Sham rats exhibited normal structure with no histopathological changes observed. However, lung tissue from CLP rats had more extensive leukocyte infiltration (arrows) and alveolar flooding (#) than those of the Sham group (Fig. 6E). Pulmonary edema was also significantly increased in CLP rats compared to Sham rats. Wet/dry ratio was 4.2 ± 0.2 in Sham and 5.4 ± 0.3 in CLP (*P* < 0.05, Fig. 6F).

## Discussion

Because of their high numbers and sensitivity to environmental changes, platelets can function as sentinels in the body's circulation. It is widely postulated that platelets are one of the first cells to sense changes during the development of sepsis and may be the first cells to respond to this derangement (de Stoppelaar et al. 2014). The response of platelets could orchestrate both the coagulation and inflammatory responses that are altered, and thus may regulate a number of pathways that are pathological and contribute to the progression of disease during sepsis. Increased platelet activation occurs in sepsis and is positively associated with increased sepsis severity (Gawaz et al. 1997; Russwurm et al. 2002; de Stoppelaar et al. 2014; Greco et al. 2017; Layios et al. 2017). However, the potential mechanisms by which platelets may contribute to multliorgan failure in sepsis remains unclear. In this study, we used a rat model of chronic sepsis induced by CLP to determine if NLRP3 is activated in platelets in response to sepsis. We also sought to correlate platelet activation with renal and pulmonary injury.

The NLRP3 inflammasome is composed of the NLRP3 protein, apoptosis‐associated speck‐like protein (ASC) and procaspase‐1. This complex catalyzes the conversion of procaspase 1 to caspase 1, which contributes to the production and secretion of IL‐1*β* and IL‐18 (Latz et al. 2013; Shao et al. 2015). NLRP3 activation is linked to a number of inflammatory conditions, including sepsis (Long et al. 2016); and inhibition of NLRP3 is protective against sepsis‐induced lung injury (Luo et al. 2014) and shock (Mao et al. 2013) in mice. However, very few studies have examined NLRP3 activation in platelets (Hottz et al. 2013; Murthy et al. 2017). This study is the first to examine platelet NLRP3 activation in sepsis. We postulate platelet NLRP3 activation to be relevant not only to the understanding of the mechanisms of sepsis‐induced organ dysfunction, but also a novel therapeutic target.

Platelet activation was significantly increased in response to in vitro LPS activation and after 72 h in CLP rats. We also demonstrated, for the first time, that NLRP3 is activated in platelets in response to both LPS stimulation in vitro and in vivo CLP through demonstration of NLRP3 and ASC colocalization (Fig. 2), increased caspase 1 activity (Fig. 3), and increased IL‐1*β* secretion (Fig. 3) from CLP platelets compared to Sham platelets. CLP rats demonstrated increased circulating and tissue levels of NLRP3‐associated cytokines IL‐1*β* and IL‐18. This also correlated with increased renal and pulmonary injury in CLP rats compared to Sham controls. Thus platelets may potentially contribute to the development of renal and pulmonary injury in sepsis through modulation of the immune response through NLRP3 associated cytokines.

IL‐1*β* is a proinflammatory cytokine known to induce endothelial permeability and compromise barrier function in a number of inflammatory conditions (Dinarello 2005; Labus et al. 2014; Tossetta et al. 2014; Matsuda et al. 2015). Platelets constitutively express immature IL‐1*β* mRNA. Upon activation, platelets splice mature IL‐1*β* mRNA (Denis et al. 2005; Shashkin et al. 2008). More importantly, IL‐1*β* secretion by platelets has been shown to mediate increased endothelial permeability after incubation with Dengue virus in vitro (Hottz et al. 2013). IL‐18 is a pleiotropic cytokine that has been shown to contribute to renal infiltration of CD4^+^T cells, macrophages, and neutrophils resulting in tubular injury and necrosis in ischemia–reperfusion injury associated AKI (He et al. 2008; Wu et al. 2008; Yano et al. 2015), though its role in sepsis remains unclear. Interestingly, recent studies show that platelets secrete and are the main source of IL‐18 in the circulation (Allam et al. 2012).

NLRP3 is a member of a family of protein complexes in the immune system critical for modulation of both innate and adaptive immunity. NLRP3 activation is linked to a number of inflammatory conditions, including sepsis (Long et al. 2016). Only two other studies have shown NLRP3 activation in platelets (Hottz et al. 2013; Murthy et al. 2017). Systemic inhibition of NLRP3 is protective against sepsis‐induced renal and pulmonary injury in mouse models of sepsis and shock (Mao et al. 2013; Luo et al. 2014). NLRP3‐deficient mice showed increased survival which was attributed to decrease autophagy and increased phagocytosis by neutrophils (Jin et al. 2017). We show for the first time that NLRP3 is activated in platelets stimulated by LPS or sepsis in CLP rats. The revelation of NLRP3 activation in platelets in sepsis may uncover additional mechanisms that could contribute to improved outcomes in sepsis associated with NLRP3 inhibition.

A number of preclinical studies have demonstrated improvements in organ injury and function as well as mortality with antiplatelet therapy in various animal models of sepsis (Gawaz et al. 1997; de Stoppelaar et al. 2014). Results from clinical studies have been less consistent. However, the majority of studies showed reduced mortality, shorter hospital stay, or reduced incidence of AKI and ALI in critically ill patients (Russwurm et al. 2002; Winning et al. 2010; Erlich et al. 2011; Kor et al. 2011; de Stoppelaar et al. 2014). While most of these studies were retrospective, the recent PLATelet inhibition and patient Outcomes (PLATO) trial showed a reduction in adverse events in patients on antiplatelet therapy (Storey et al. 2014).

While associations with platelet activation and sepsis severity are clearly demonstrated clinically, and both preclinical and clinical studies show improved outcomes with antiplatelet therapy in sepsis, a knowledge gap still remains in our understanding of how platelets cause progression in sepsis. This study suggests that NLRP3 may be a mechanism by which platelets regulate the exaggerated inflammation that occurs in sepsis. Elucidation of mechanisms downstream of platelet NLRP3 activation may increase our understanding of sepsis pathophysiology and lead to the development of novel therapeutic strategies to improve sepsis outcomes.

## Conflict of Interest

The authors have no conflicts of interest to disclose.

## Supporting information




**Figure S1.** Light microscopy of Sham and CLP platelets representative light microscopy images of platelets from Sham and CLP rats.

## References

[phy214073-bib-0001] Abderrazak, A. , T. Syrovets , D. Couchie , K. El Hadri , B. Friguet , T. Simmet , et al. 2015 NLRP3 inflammasome: from a danger signal sensor to a regulatory node of oxidative stress and inflammatory diseases. Redox. Biol. 4:296–307. 25625584 10.1016/j.redox.2015.01.008 PMC4315937

[phy214073-bib-0002] Allam, O. , S. Samarani , R. Marzouk , and A. Ahmad . 2012 Human platelets produce and constitute the main source of IL‐18 in the circulation (44.25). J. Immunol. 188:44.25.

[phy214073-bib-0003] Alobaidi, R. , R. K. Basu , S. L. Goldstein , and S. M. Bagshaw . 2015 Sepsis‐associated acute kidney injury. Semin. Nephrol. 35:2–11. 25795495 10.1016/j.semnephrol.2015.01.002 PMC4507081

[phy214073-bib-0004] Denis, M. M. , N. D. Tolley , M. Bunting , H. Schwertz , H. Jiang , S. Lindemann , et al. 2005 Escaping the nuclear confines: signal‐dependent pre‐mRNA splicing in anucleate platelets. Cell 122:379–391. 16096058 10.1016/j.cell.2005.06.015 PMC4401993

[phy214073-bib-0005] Dinarello, C. A. 2005 Interleukin‐1beta. Crit. Care Med. 33(12 Suppl):S460–S462. 16340421 10.1097/01.ccm.0000185500.11080.91

[phy214073-bib-0006] Erlich, J. M. , D. S. Talmor , R. Cartin‐Ceba , O. Gajic , and D. J. Kor . 2011 Prehospitalization antiplatelet therapy is associated with a reduced incidence of acute lung injury: a population‐based cohort study. Chest 139:289–295. 20688925 10.1378/chest.10-0891 PMC3032365

[phy214073-bib-0007] Gawaz, M. , T. Dickfeld , C. Bogner , S. Fateh‐Moghadam , and F. J. Neumann . 1997 Platelet function in septic multiple organ dysfunction syndrome. Intensive Care Med. 23:379–385. 9142575 10.1007/s001340050344

[phy214073-bib-0008] Gong, Z. , J. Zhou , H. Li , Y. Gao , C. Xu , S. Zhao , et al. 2015 Curcumin suppresses NLRP3 inflammasome activation and protects against LPS‐induced septic shock. Mol. Nutr. Food Res. 59:2132–2142. 26250869 10.1002/mnfr.201500316

[phy214073-bib-0009] Greco, E. , E. Lupia , O. Bosco , B. Vizio , and G. Montrucchio . 2017 Platelets and multi‐organ failure in sepsis. Int. J. Mol. Sci. 18:2200. 10.3390/ijms18102200 PMC5666881 29053592

[phy214073-bib-0010] He, Z. , L. Lu , C. Altmann , T. S. Hoke , D. Ljubanovic , A. Jani , et al. 2008 Interleukin‐18 binding protein transgenic mice are protected against ischemic acute kidney injury. Am. J. Physiol. Renal Physiol. 295:F1414–F1421. 18753296 10.1152/ajprenal.90288.2008 PMC2584896

[phy214073-bib-0011] Hottz, E. D. , J. F. Lopes , C. Freitas , R. Valls‐de‐Souza , M. F. Oliveira , M. T. Bozza , et al. 2013 Platelets mediate increased endothelium permeability in dengue through NLRP3‐inflammasome activation. Blood 122:3405–3414. 24009231 10.1182/blood-2013-05-504449 PMC3829114

[phy214073-bib-0012] Hubbard, W. J. , M. Choudhry , M. G. Schwacha , J. D. Kerby , L. W. III Rue , K. I. Bland , et al. 2005 Cecal ligation and puncture. Shock 24:52–57. 16374373 10.1097/01.shk.0000191414.94461.7e

[phy214073-bib-0013] Jin, L. , S. Batra , and S. Jeyaseelan . 2017 Deletion of Nlrp3 augments survival during polymicrobial sepsis by decreasing autophagy and enhancing phagocytosis. J. Immunol. 198:1253–1262. 28031338 10.4049/jimmunol.1601745 PMC5263118

[phy214073-bib-0014] Kalbitz, M. , F. Fattahi , J. J. Grailer , L. Jajou , E. A. Malan , F. S. Zetoune , et al. 2016 Complement‐induced activation of the cardiac NLRP3 inflammasome in sepsis. FASEB J. 30:3997–4006. 27543123 10.1096/fj.201600728R PMC5102118

[phy214073-bib-0015] Kaya, O. , Y. S. Koca , I. Barut , S. Baspinar , and M. Z. Sabuncuoglu . 2015 L‐carnitine reduces acute lung injury in experimental biliary obstruction. Saudi Med. J. 36:1046–1052. 26318460 10.15537/smj.2015.9.12206 PMC4613627

[phy214073-bib-0016] Kor, D. J. , J. Erlich , M. N. Gong , M. Malinchoc , R. E. Carter , O. Gajic , et al. 2011 Association of pre‐hospitalization aspirin therapy and acute lung injury: results of a multicenter international observational study of at‐risk patients. Crit. Care Med. 39:2393. 21725238 10.1097/CCM.0b013e318225757f PMC3196806

[phy214073-bib-0017] Labus, J. , S. Häckel , L. Lucka , and K. Danker . 2014 Interleukin‐1beta induces an inflammatory response and the breakdown of the endothelial cell layer in an improved human THBMEC‐based in vitro blood‐brain barrier model. J. Neurosci. Methods 228:35–45. 24631939 10.1016/j.jneumeth.2014.03.002

[phy214073-bib-0018] Latz, E. , T. S. Xiao , and A. Stutz . 2013 Activation and regulation of the inflammasomes. Nat. Rev. Immunol. 13:397–411. 23702978 10.1038/nri3452 PMC3807999

[phy214073-bib-0019] Layios, N. , C. Delierneux , A. Hego , J. Huart , C. Gosset , C. Lecut , et al. 2017 Sepsis prediction in critically ill patients by platelet activation markers on ICU admission: a prospective pilot study. Intensive Care Med. Exp. 2017:32. 10.1186/s40635-017-0145-2 PMC5505890 28699088

[phy214073-bib-0020] Long, H. , B. Xu , Y. Luo , and K. Luo . 2016 Artemisinin protects mice against burn sepsis through inhibiting NLRP3 inflammasome activation. Am. J. Emerg. Med. 34:772–777. 26830216 10.1016/j.ajem.2015.12.075

[phy214073-bib-0021] Luo, Y. P. , L. Jiang , K. Kang , D. S. Fei , X. L. Meng , C. C. Nan , et al. 2014 Hemin inhibits NLRP3 inflammasome activation in sepsis‐induced acute lung injury, involving heme oxygenase‐1. Int. Immunopharmacol. 20:24–32. 24583148 10.1016/j.intimp.2014.02.017

[phy214073-bib-0022] Mao, K. , S. Chen , M. Chen , Y. Ma , Y. Wang , B. Huang , et al. 2013 Nitric oxide suppresses NLRP3 inflammasome activation and protects against LPS‐induced septic shock. Cell Res. 23:201. 23318584 10.1038/cr.2013.6 PMC3567828

[phy214073-bib-0023] Matsuda, S. , T. Fujita , M. Kajiya , K. Kashiwai , K. Takeda , H. Shiba , et al. 2015 Brain‐derived neurotrophic factor prevents the endothelial barrier dysfunction induced by interleukin‐1β and tumor necrosis factor‐α. J. Periodontal Res. 50:444–451. 25203938 10.1111/jre.12226

[phy214073-bib-0024] Moon, J. S. , S. Lee , M. A. Park , I. I. Siempos , M. Haslip , P. J. Lee , et al. 2015 UCP2‐induced fatty acid synthase promotes NLRP3 inflammasome activation during sepsis. J. Clin. Invest. 125:665–680. 25574840 10.1172/JCI78253 PMC4319445

[phy214073-bib-0025] Murthy, P. , F. Durco , J. L. Miller‐Ocuin , T. Takedai , S. Shankar , X. Liang , et al. 2017 The NLRP3 inflammasome and bruton's tyrosine kinase in platelets co‐regulate platelet activation, aggregation, and in vitro thrombus formation. Biochem. Biophys. Res. Commun. 483:230–236. 28034752 10.1016/j.bbrc.2016.12.161

[phy214073-bib-0026] Russwurm, S. , J. Vickers , A. Meier‐Hellmann , P. Spangenberg , D. Bredle , K. Reinhart , et al. 2002 Platelet and leukocyte activation correlate with the severity of septic organ dysfunction. Shock 17:263–268. 11954824 10.1097/00024382-200204000-00004

[phy214073-bib-0027] Shao, B. Z. , Z. Q. Xu , B. Z. Han , D. F. Su , and C. Liu . 2015 NLRP3 inflammasome and its inhibitors: a review. Front. Pharmacol. 6:262. 26594174 10.3389/fphar.2015.00262 PMC4633676

[phy214073-bib-0028] Shashkin, P. N. , G. T. Brown , A. Ghosh , G. K. Marathe , and T. M. McIntyre . 2008 Lipopolysaccharide is a direct agonist for platelet RNA splicing. J. Immunol. 181:3495–3502. 18714022 10.4049/jimmunol.181.5.3495 PMC2551315

[phy214073-bib-0029] de Stoppelaar, S. F. , C. van't Veer , and T. van der Poll . 2014 The role of platelets in sepsis. Thromb. Haemost. 112:666–677. 24966015 10.1160/TH14-02-0126

[phy214073-bib-0030] Storey, R. F. , S. K. James , A. Siegbahn , C. Varenhorst , C. Held , J. Ycas , et al. 2014 Lower mortality following pulmonary adverse events and sepsis with ticagrelor compared to clopidogrel in the PLATO study. Platelets 25:517–525. 24127651 10.3109/09537104.2013.842965 PMC4220996

[phy214073-bib-0031] Tossetta, G. , F. Paolinelli , C. Avellini , E. Salvolini , P. Ciarmela , T. Lorenzi , et al. 2014 IL‐1beta and TGF‐beta weaken the placental barrier through destruction of tight junctions: an in vivo and in vitro study. Placenta 35:509–516. 24768095 10.1016/j.placenta.2014.03.016

[phy214073-bib-0032] Umbro, I. , G. Gentile , F. Tinti , P. Muiesan , and A. P. Mitterhofer . 2016 Recent advances in pathophysiology and biomarkers of sepsis‐induced acute kidney injury. J. Infect. 72:131–142. 26702738 10.1016/j.jinf.2015.11.008

[phy214073-bib-0033] Vincent, J. L. , D. R. Nelson , and M. D. Williams . 2011 Is worsening multiple organ failure the cause of death in patients with severe sepsis? Crit. Care Med. 39:1050–1055. 21317650 10.1097/CCM.0b013e31820eda29

[phy214073-bib-0034] Wang, P. , J. Huang , Y. Li , R. Chang , H. Wu , J. Lin , et al. 2015 Exogenous carbon monoxide decreases sepsis‐induced acute kidney injury and inhibits NLRP3 inflammasome activation in rats. Int. J. Mol. Sci. 16:20595–20608. 26334271 10.3390/ijms160920595 PMC4613220

[phy214073-bib-0035] Winning, J. , J. Neumann , M. Kohl , R. A. Claus , K. Reinhart , M. Bauer , et al. 2010 Antiplatelet drugs and outcome in mixed admissions to an intensive care unit. Crit. Care Med. 38:32–37. 19770746 10.1097/CCM.0b013e3181b4275c

[phy214073-bib-0036] Wu, H. , M. L. Craft , P. Wang , K. R. Wyburn , G. Chen , J. Ma , et al. 2008 IL‐18 contributes to renal damage after ischemia‐reperfusion. J. Am. Soc. Nephrol. 19:2331–2341. 18815244 10.1681/ASN.2008020170 PMC2588109

[phy214073-bib-0037] Yano, T. , Y. Nozaki , K. Kinoshita , S. Hino , Y. Hirooka , K. Niki , et al. 2015 The pathological role of IL‐18Ralpha in renal ischemia/reperfusion injury. Lab. Invest. 95:78–91. 25329004 10.1038/labinvest.2014.120

