# Supplementary figures and images for: NLRP3 inflammasome activation in platelets in response to sepsis
Source: Physiol Rep. 2019 May 3;7(9):e14073. doi: 10.14814/phy2.14073 (PMC6499866; doi:10.14814/phy2.14073)

# Supplemental Figure 1

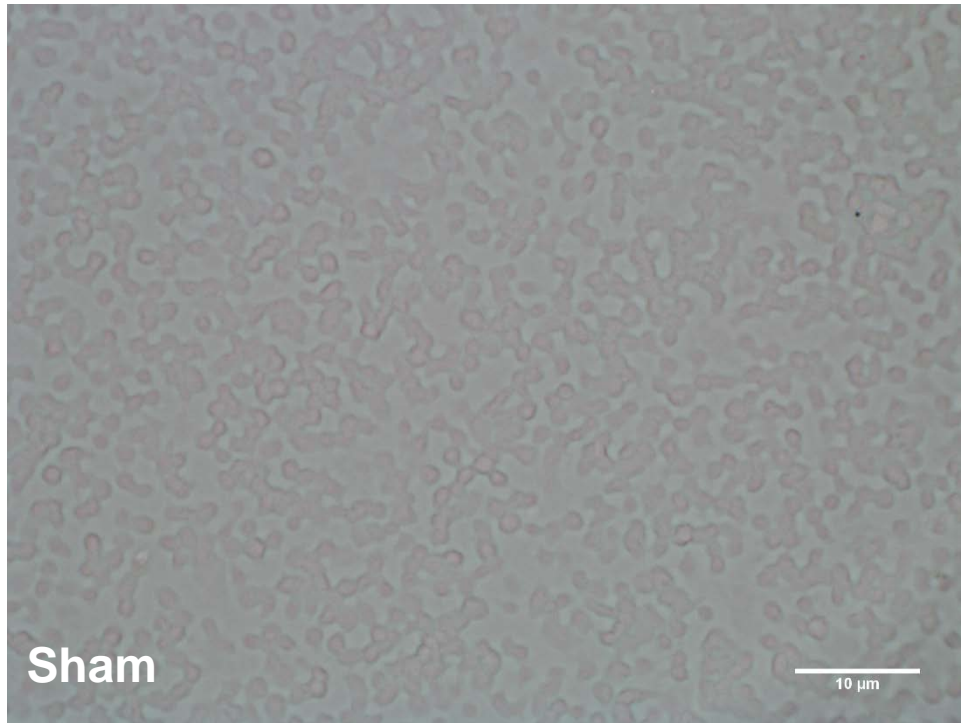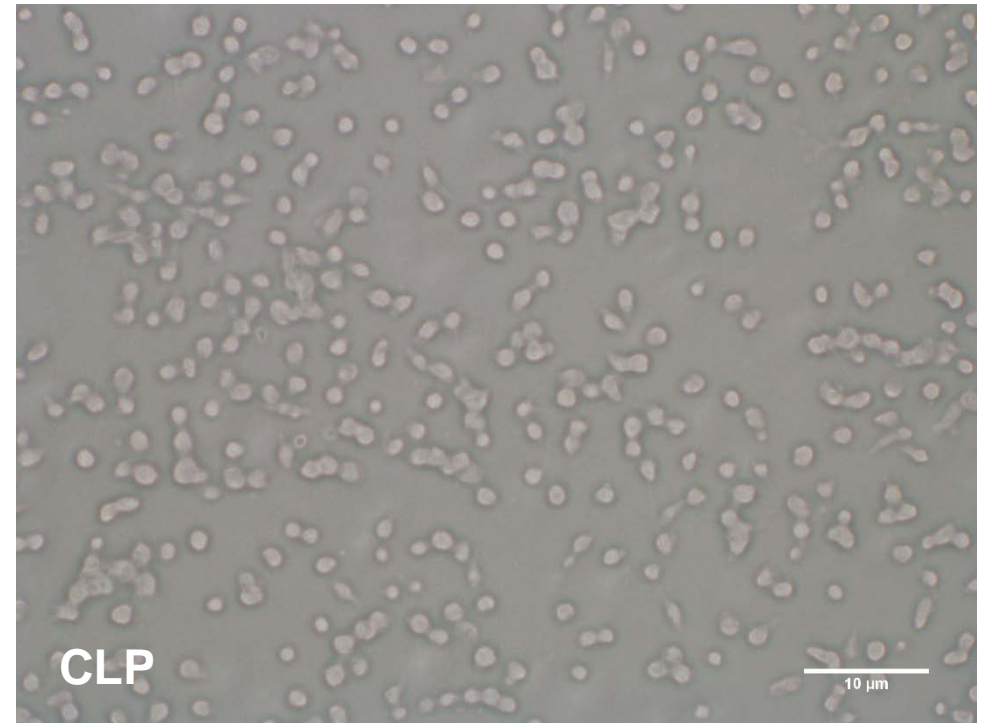

Supplement: Supplementary file 1 — Figure S1. Light microscopy of Sham and CLP platelets representative light microscopy images of platelets from Sham and CLP rats. [file PHY2-7-e14073-s001.pdf]
